# Salt Stress Induced Changes in Photosynthesis and Metabolic Profiles of One Tolerant (‘Bonica’) and One Sensitive (‘Black Beauty’) Eggplant Cultivars (*Solanum melongena* L.)

**DOI:** 10.3390/plants11050590

**Published:** 2022-02-22

**Authors:** Sami Hannachi, Kathy Steppe, Mabrouka Eloudi, Lassaad Mechi, Insaf Bahrini, Marie-Christine Van Labeke

**Affiliations:** 1 Department of Biology, College of Science, University of Hail, P.O. Box 2440, Hail 81451, Saudi Arabia; insafafa@yahoo.fr; 2 Department of Plants and Crops, Faculty of Bioscience Engineering, Ghent University, Coupure Links, 653, 9000 Ghent, Belgium; kathy.steppe@ugent.be (K.S.); mariechristine.vanlabeke@ugent.be (M.-C.V.L.); 3 Department of Chemistry, College of Science, University of Hail, P.O. Box 2440, Hail 8145, Saudi Arabia; doc_mo@ymail.com (M.E.); mechilassaad@yahoo.fr (L.M.)

**Keywords:** antioxidant response, electrolyte leakage, mineral content, photosynthesis, photorespiration, total phenols, salt, *Solanum melongena* L.

## Abstract

The impact of salinity on the physiological and biochemical parameters of tolerant (‘Bonica’) and susceptible (‘Black Beauty’) eggplant varieties (*Solanum melongena* L.) was determined. The results revealed that the increase in salinity contributes to a significant decline in net photosynthesis (A_n_) in both varieties; however, at the highest salt concentration (160 mM NaCl), the decrease in photorespiration (R_l_) was less pronounced in the tolerant cultivar ‘Bonica’. Stomatal conductance (g_s_) was significantly reduced in ‘Black Beauty’ following exposure to 40 mM NaCl. However, g_s_ of ‘Bonica’ was only substantially reduced at the highest level of NaCl (160 mM). In addition, a significant decrease in Chl*a*, Chl*b*, total Chl, Chl*a/b* and carotenoids (*p* > 0.05) was found in ‘Black Beauty’, and soluble carbohydrates accumulation and electrolyte leakage (EL) were more pronounced in ‘Black Beauty’ than in ‘Bonica’. The total phenols increase in ‘Bonica’ was 65% higher than in ‘Black Beauty’. In ‘Bonica’, the roots displayed the highest enzyme scavenging activity compared to the leaves. Salt stress contributes to a significant augmentation of root catalase and guaiacol peroxidase activities. In ‘Bonica’, the Na concentration was higher in roots than in leaves, whereas in ‘Black Beauty‘, the leaves accumulated more Na. Salt stress significantly boosted the Na/K ratio in ‘Black Beauty’, while no significant change occurred in ‘Bonica’. ACC deaminase activity was significantly higher in ‘Bonica’ than in ‘Black Beauty’.

## 1. Introduction

Salinity is one of the most serious abiotic stresses limiting plant growth and development, especially in salt-sensitive crops [1,2]. The detrimental effect of high salinity on plants can be observed at the whole-plant level as the death of the plant and/or decreases in productivity. Many plants develop mechanisms either to exclude salt from their cells or to tolerate its presence within the cells. During the onset and development of salt stress within a plant, all major processes such as photosynthesis, protein synthesis, and energy and lipid metabolism are affected. The earliest response is a reduction in the rate of leaf surface expansion, followed by a cessation of expansion [3] as the stress intensifies. Salt accumulation in soil solution reduces water and nutrient uptake. This leads to osmotic stress, ion toxicity, nutrient imbalances and a water deficit. Excessive concentrations of salt ions also injure photosynthetically active leaves, and may lead to chlorosis and early leaf senescence [4]. In addition, the supply of carbohydrates, which are needed for cell growth, may be hampered because photosynthesis rates are usually lower in plants exposed to salinity, and especially to sodium chloride (NaCl) [5,6].

The onset of salt stress and its intensification affect the efficiency of photosynthesis, stomatal conductance and accelerate plant leaf senescence [7]. The salt-induced reduction in photosynthesis rate can be caused by partial stomatal closure caused by associated osmotic stress [8,9,10], by non-stomatal limitations caused by an excessive salt build-up and/or ionic imbalance in the leaves [11,12] or by both limitations [13,14]. The authors of [15] emphasized that compatible solutes accumulation is necessary to correct the ionic imbalance and the potential water decrease. When NaCl directly hampers leaves and roots the plant will likely suffer from oxidative stress. Oxidative stress generated by an excessive NaCl accumulation affects the integrity of plant cellular membranes [16,17,18], causes electrolyte leakage, and damages chloroplasts [19,20]. Salinity tolerance is highly related to the maintenance of net photosynthetic rates [21], stomatal conductance [22,23] and elevated chlorophyll concentration [24,25]. Excess salt leads to a change in the ionic composition of the stroma of the chloroplasts, which in turn can cause shrinkage of the thylakoids and stacking of adjacent membranes in grana [26]. An irreversible impairment of the photosynthetic apparatus, associated with a reduction in Rubisco activity, occurs when the stress is prolonged and salt continues to accumulate in the leaves [27,28]. As proposed by [29,30] under drought stress, the reduction in O_2_ in the chloroplasts via the Mehler-peroxidase pathway and possible photorespiration might provide photo-protection by acting as an alternative sink for excess energy in the photosynthetic apparatus. Photorespiration may thus be an alternative sink for light-induced electron flow, and it is often presented as a process that may help consume an appreciable electron flow during periods of restricted CO_2_ availability in the chloroplasts and high irradiance [31,32]. According to [33], the ratio J_C_/J_O_ (photosynthetic electron flux density used for RuBP carboxylation/photosynthetic electron flux density used for RuBP oxygenation) is a good indicator of relative rates of carboxylation versus oxygenation and may be directly controlled by the kinetic properties of Rubisco. However, no data on the role of this alterative sink under stress conditions are available in eggplants.

The overproduction of scavenging reactive oxygen species (ROS) in plant cells under stress contributes to the damage of cellular components, including DNA, proteins and membrane lipids [17,34]. To scavenge ROS, plants have evolved both enzymatic and non-enzymatic defense systems [34,35,36]. As emphasized in previous studies [34,37], over the course of evolution, the enzymatic antioxidant defense system developed by plant cells has included several enzymes such as SOD, APX, GPX, GR, CAT, etc.; SOD converts superoxide to H_2_O_2_ [38]. This H_2_O_2_ is scavenged by CAT and other peroxidases (APX, POD) to H_2_O [39].

In addition to these internal plant processes, salinity can also be counteracted by using plant growth-promoting bacteria (PGPB) which live in the rhizosphere and rhizoplane of the plants [40]. PGPB contain several beneficial bacteria that contribute to improving plant salt tolerance by reducing the ethylene biosynthesis via hydrolysis of 1-aminocyclopropane-1-carboxylic acid (ACC) by the ACC deaminase enzyme and converting it into ammonia and α-ketobutyrate. ACC is the direct precursor of the hormone ethylene in plants [41]. Consequently, PGPB with ACC deaminase activity lower the abiotic stress generated by ethylene synthesis and its effect on plants [42].

This study aims to evaluate the impact of increasing salinity on the photosynthetic machinery of a salt-tolerant eggplant variety (*Solanum melongena* L.) ‘Bonica’ and a salt-susceptible variety ‘Black Beauty’. Moreover, we investigated the biochemical responses to salt stress through selected parameters such as chlorophyll content, electrolyte leakage (EL), total phenols and carbohydrate accumulation patterns, the activity of three antioxidant enzymes (CAT, APX, and POD), sodium and chloride accumulation. In a separate experiment ACC deaminase-producing bacteria were isolated from roots of ‘Black Beauty’ and ‘Bonica’ and their ACC deaminase activity was determined to investigate if they could mitigate the effects of salt stress.

## 2. Results

### 2.1. NaCl Induced Changes in Gas Exchange Parameters

**Figure 1:**
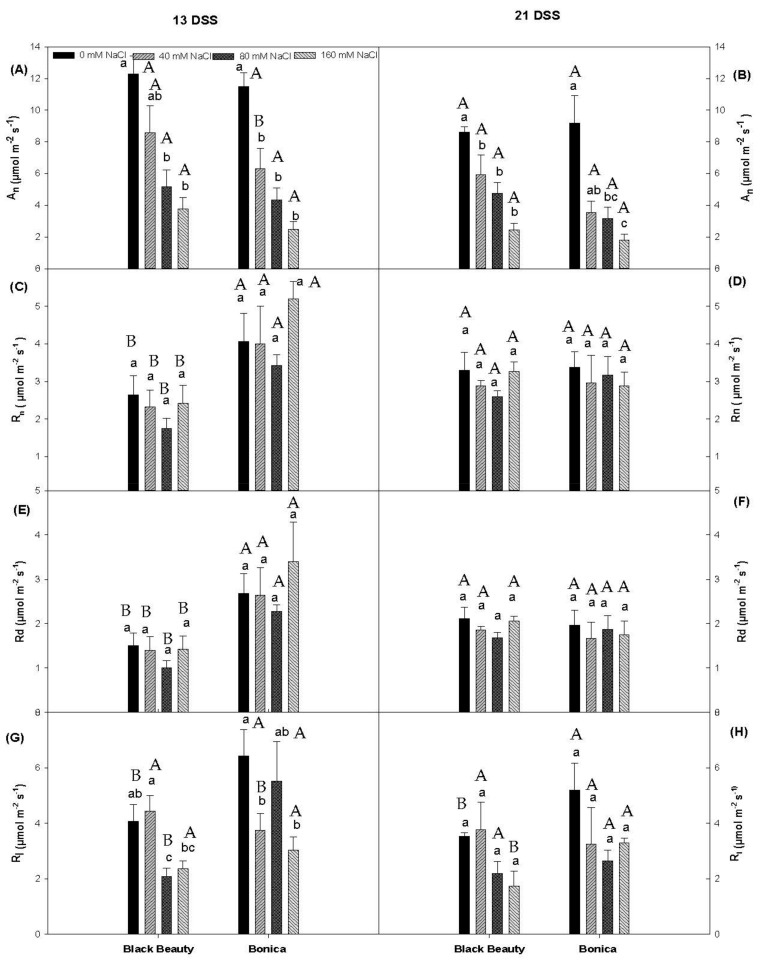
Changes in A_n_ (net photosynthesis) after 13DSS (**A**) and 21DSS (**B**), R_n_ (mitochondrial respiration during the night) after 13DSS (**C**) and 21DSS (**D**), R_d_ (mitochondrial respiration during the day) after 13DSS (**E**) and 21DSS (**F**) and R_l_ (photorespiration) after 13DSS (**G**) and 21DSS (**H**) in leaves of eggplant varieties (‘Black Beauty’ and ‘Bonica’) under different salt levels (date 1: 13 June 2012; date 2 = 21 June 2012). Data are means ± SE of four replicates. Significant dissimilarities are shown by different lower-case letters between treatments and by capital letters between varieties (*p* = 0.05), based on Tukey’s HSD test.

The impact of increasing salt concentration on photosynthetic machinery, over time, is shown in Figure 1. Salt stress significantly reduced A_n_ in each variety, at 13 days of salt stress (DSS) (Figure 1A). The highest salt concentration caused a decrease in A_n_ of 69.2% in ‘Black Beauty’ and 78.4% in ‘Bonica’ compared to their respective controls (Figure 1A). The same response occurred in both varieties at 21 DSS (Figure 1B). Increasing salinity did not significantly change the traditional dark respiration during the night (R_n_) and the mitochondrial respiration during the day (R_d_) in both cultivars after 13 and 21 DSS (Figure 1C–F). Overall, under salinity conditions, R_d_ (*p* = 0.001) and R_n_ (*p* = 0.003) were higher in ‘Bonica’ than ‘Black Beauty’ at 13 DSS (Figure 1C,E). Imposing salt stress over thirteen days caused a significant reduction in R_l_ in each variety with 160 mM NaCl at the highest salt level, the decrease in R_l_ being more accentuated in ‘Black Beauty’ than in ’Bonica‘ (Figure 1G). When subjected to salt stress for 21 days, ‘Black Beauty’ showed a lower R_l_ than ‘Bonica’ for the highest (160 mM NaCl) salt concentration (*p* = 0.002; Figure 1H).

**Figure 2:**
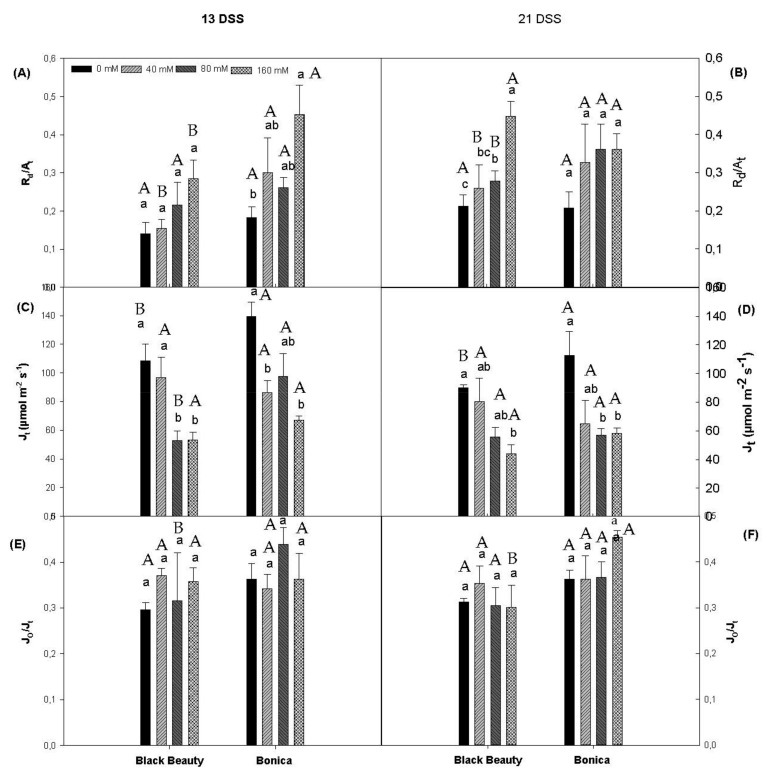
Changes in R_d_/A_t_ (mitochondrial respiration during the day/total assimilation rate) after 13DSS (**A**) and 21DSS (**B**), in J_t_ (total electron flow) after 13DSS (**C**) and 21DSS (**D**), and in J_o_/J_t_ (photosynthetic electron flux density used for RuBP oxygenation/photosynthetic electron flux density used for RuBP carboxylation and oxygenation) after 13DSS (**E**) and 21DSS (**F**) in leaves of eggplant varieties (‘Black Beauty’ and ‘Bonica’) under different salinity levels on two measuring dates (date 1: 13 June 2012; date 2 = 21 June 2012). Data are means ± SE of four replicates. Significant dissimilarities are shown by different lower-case letters between treatments and by different capital letters between varieties (*p* = 0.05), based on Tukey’s HSD test.

The increasing salt concentration enhanced the ratio R_d_/A_t_ (A_t_: total assimilation rate) in each variety and for both measuring dates (13 DSS and 21 DSS). This increase was less accentuated in ‘Black Beauty’ than in ‘Bonica’ after thirteen days of salt stress. However, at 21 DSS, the R_d_/A_t_ ratio was more elevated in ‘Black Beauty’ and became stable in ‘Bonica’ (Figure 2A,B). Salt stress significantly decreased the total electron flow (J_t_) in ‘Black Beauty’ (*p* = 0.003) and ‘Bonica’ (*p* = 0.003) from 13 DSS on. The highest salt concentration (160 mM of NaCl) caused reductions in J_t_ of 50.8% and 51.9%, respectively, in ‘Black Beauty’ and ‘Bonica’ compared to their respective controls (Figure 2C). Moreover, after 21 days of salt stress, J_t_ declined by 51.3% (*p* = 0.017) and 48.2% (*p* = 0.021), respectively, in ‘Black Beauty’ and ‘Bonica’ for 160 mM of NaCl compared to their respective controls (Figure 2D). The ratio J_o_/J_t_ was estimated to investigate the relative importance of the photorespiratory pathway as a strategy for dissipating the overabundant energy (Figure 2E,F). After thirteen days of salt stress, no significant impact on J_o_/J_t_ was observed in ‘Black Beauty’ and ‘Bonica’. Overall, ‘Black Beauty’ and ‘Bonica’ utilized less than 40% of the total electron flow for oxygenation of RuBP (Figure 2E,F).

### 2.2. NaCl Induced Changes in Plant Water Relations

**Figure 3:**
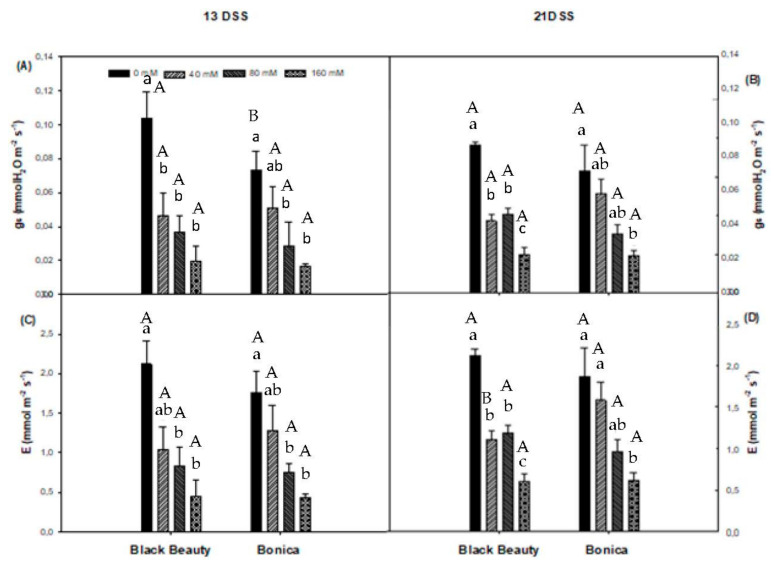
Changes in g_s_ (stomatal conductance to water vapor) after 13DSS (**A**) and 21DSS (**B**) and in E (transpiration) after 13DSS (**C**) and 21DSS (**D**) in leaves of the eggplant varieties (‘Bonica’ and ‘Black Beauty’) under different salinity levels on two measuring dates (date 1 = 13 June 2012, date 2 = 23 June 2012). Data are means ± SE of four replicates. Significant dissimilarities are shown by different lower-case letters between treatments and by different capital letters between varieties (*p* = 0.05) based on Tukey’s HSD test.

**Figure 4:**
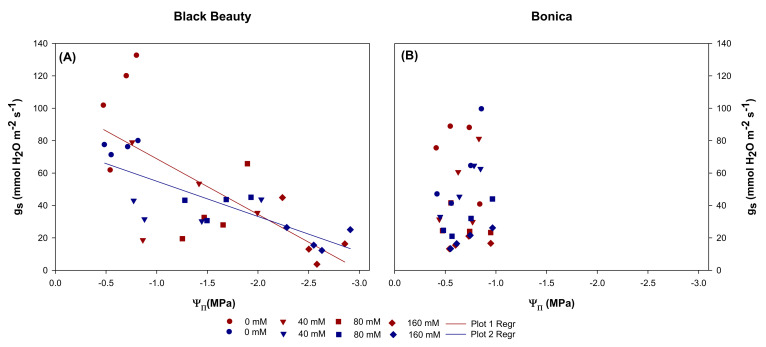
Relationship between g_s_ (stomatal conductance to water vapor) and ψ_π_ (osmotic potential) measured after 13 DSS (red color) and after 21 DSS (blue color) in ‘Black Beauty’ (**A**) and ‘Bonica’ (**B**).

Under salinity conditions, the stomatal conductance to water vapor (g_s_) significantly decreased in both varieties. A moderate salt concentration (40 mM NaCl) contributed already to a significant decline in g_s_ in ‘Black Beauty’ for both measuring dates. In ‘Bonica’, a significant reduction in g_s_ occurred only at the highest salinity concentration (160 mM NaCl). Thirteen days of salt stress caused decreases in g_s_ of 81% and 78%, respectively, in ‘Black Beauty’ and ‘Bonica’ at 160 mM NaCl relative to their controls (Figure 3A). The same trend was observed in both cultivars at 21 DSS (Figure 3B). A close correlation between stomatal conductance and net assimilation was detected in ‘Black Beauty’(r = 0.89, *p* = 0.010) and in ‘Bonica’ (r = 0.88, *p* = 0.010) (Figure 4A,B).

Stomatal closure had a negative impact on transpiration rates (E). At 13 DSS, 80 mM NaCl caused a significant decrease in E for both varieties, whereas imposing salt stress over 21 days significantly reduced E at 40 mM NaCl in ‘Black Beauty’. The highest salinity level (160 mM NaCl) contributed to a reduction in E of 78.6% and 75.4%, respectively, in ‘Black Beauty’ and ‘Bonica’ compared to their controls at 13 DSS (Figure 3C) and of 72.1% and 67.2%, respectively, at 21 DSS in ‘Black Beauty’ and ‘Bonica’ compared to their respective controls (Figure 3D).

The rising salinity level caused a significant reduction in both midday leaf water potential (ψ_H2O_) and leaf osmotic potential (ψ_π_) in ‘Black Beauty’ and reached values of −1.9 MPa and −2.5 MPa for the highest salt level. In addition, a significant correlation was observed in ‘Black Beauty’ between g_s_ and ψ_π_: a more negative osmotic potential is closely linked to a higher stomatal closure (Figure 4). However, ‘Bonica’ succeeded to maintain a relatively steady leaf water potential. Moreover, no significant change was observed in the osmotic potential of leaves by increasing NaCl concentration in ‘Bonica’ (Section 2.6). Additionally, g_s_ and ψ_π_ were not correlated for ‘Bonica’ (r = 0.347, *p* = 0.59).

A similar trend was observed for both cultivars when we plotted g_s_ versus ψ_H2O_ for both measurement dates (data not shown).

### 2.3. NaCl Induced Changes in Pigments and Metabolites

No significant effects on Chl*a*, Chl*b*, total Chl, Chl*a/b* and carotenoids (*p* > 0.05) were found in ‘Bonica’, whereas a significant decrease was observed for all pigments from 80 mM NaCl onwards in ‘Black Beauty’.

For the 40 mM NaCl level, we noticed a slight increase in Chl*a* in both cultivars. However, a decreasing trend for all pigments was noticed from 80 mM NaCl onwards (Table 1). As compared to the control conditions, Chl*a* decreased by 77.7% in ‘Black Beauty’ and by 22.3% in ‘Bonica’ at 160 mM NaCl, while the decline in Chl*b* between the control and the 160 mM NaCl level was 54.6% in ‘Black Beauty’ and 13.9% in ‘Bonica’. Especially at 160mM NaCl, Chl*a*, Chl*b*, and total Chl were significantly lower for ‘Black Beauty’ (Table 1).

**Figure 5:**
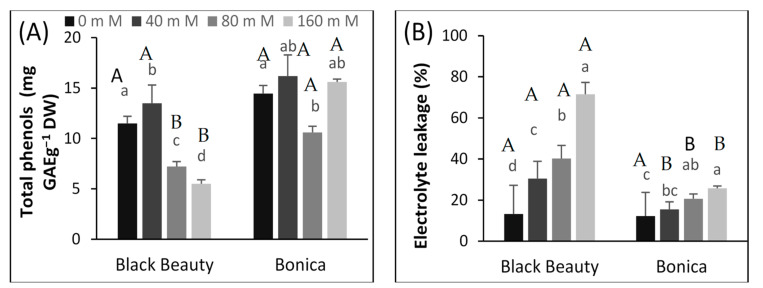
Effect of salt stress on total phenolic content (mg GAE g^−1^ DW) (**A**) and electrolyte leakage (**B**) at 30 DSS of the eggplant varieties under different salinity levels. Data are means ± SE of four replicates. Significant dissimilarities are shown by different lower-case letters between treatments and by different capital letters between varieties (*p* = 0.05) based on Tukey’s HSD test.

Increasing salt concentration contributed to a significant decrease in phenolics in both varieties. However, this decrease was less pronounced in ‘Bonica’ than in ‘Black Beauty’. A salinity level of 160 mM NaCl decreased the total phenolic compounds, respectively, 2.5-fold and 1.0-fold in ‘Bonica’ and ‘Black Beauty’ compared to controls (Figure 5A). A significant correlation between electrolyte leakage and Na+ (r = 0.80, *p*≤ 0.001) in both cultivars was observed.

Although electrolyte leakage increased significantly in both salt-stressed varieties, this increase was more pronounced in ‘Black Beauty’ than in ‘Bonica’. The highest salt concentration (160 mM NaCl) caused a 5-fold and 2-fold increase in electrolyte leakage in ‘Black Beauty’ and ‘Bonica’, respectively, compared to controls (Figure 5B).

**Figure 6:**
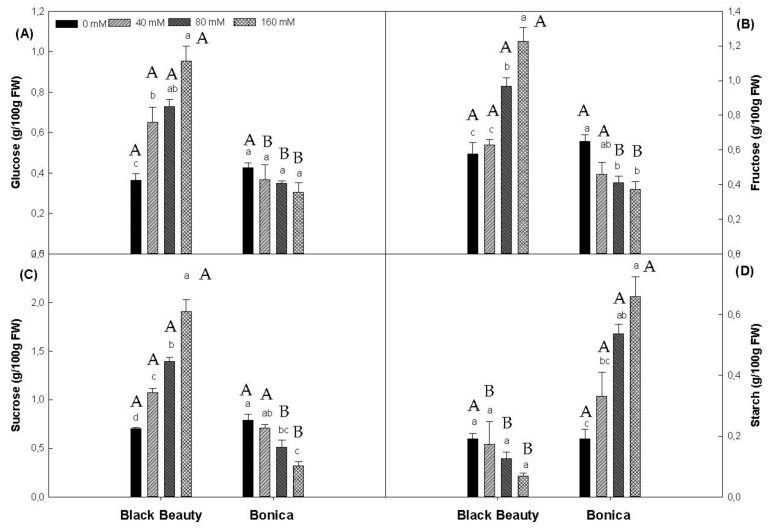
Effect of NaCl concentration on glucose (**A**), fructose (**B**), sucrose (**C**), and starch (**D**) levels in leaves of the eggplant varieties. Data are means ± SE of four replicates. Significant dissimilarities are shown by different lower-case letters between treatments and by different capital letters between varieties (*p* = 0.05) based on Tukey’s HSD test.

‘Black Beauty’ showed a significant increase in leaf glucose, fructose and sucrose content as the salinity level rose (Figure 6A–C). On the contrary, ’Bonica’ showed a significant reduction in leaf fructose and sucrose content under increasing salt stress (Figure 6B,C). Increasing salt stress led to a significant increase in starch content in ‘Bonica’ leaves, while in ‘Black Beauty’, starch content tended to decrease.

### 2.4. NaCl Induced Changes in Enzyme Activities

**Figure 7:**
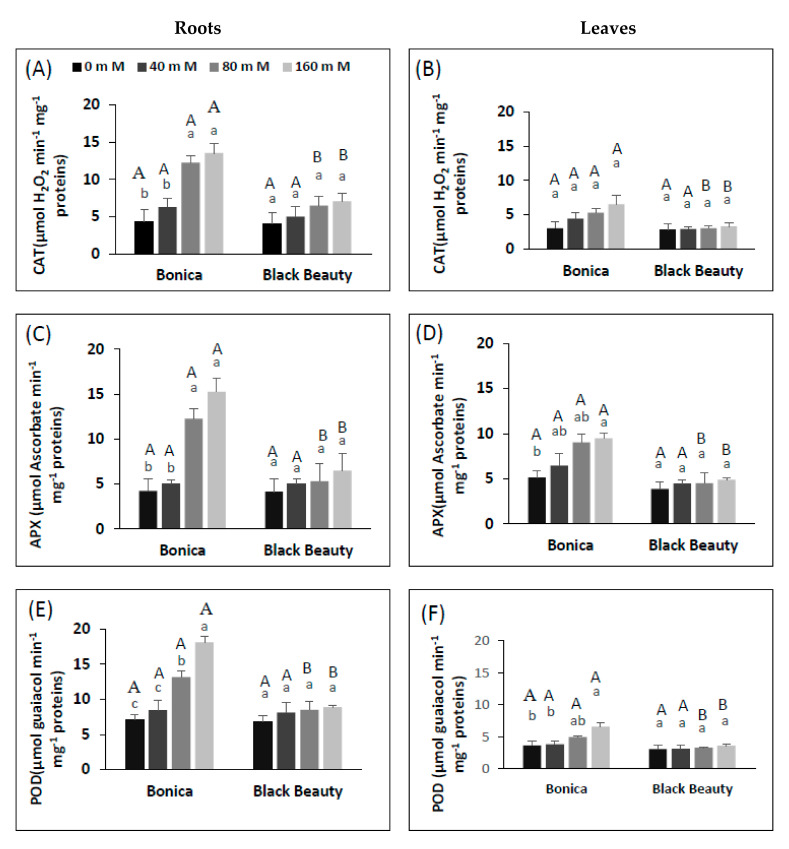
Antioxidant enzyme responses to NaCl treatments in eggplant cultivars (‘Bonica’, ‘Black Beauty’). CAT (**A**), APX (**C**) and POD (**E**) in roots. CAT (**B**), APX (**D**) and POD (**F**) in Leaves. Values are means of five replicates ± SE (*n* = 5). Significant dissimilarities are shown by different lower-case letters between treatments and by different capital letters between varieties (*p* = 0.05) based on Tukey’s HSD test.

In roots, higher salinity levels contributed to a significant increase in CAT activity in ‘Bonica’ (Figure 7A). However, in leaves, the CAT activity increase was limited and non-significant in both cultivars (Figure 7B). Compared to controls, roots CAT activity saw a 2.8- and 3.1-fold increase at salt stress levels of 80 mM and 160 mM, respectively, in ‘Bonica’, while in ‘Black Beauty’, a slight elevation of about 1.7 fold at 160 mM NaCl compared to the control was noted. Overall, the CAT activity of ‘Bonica’ was higher than that in ‘Black Beauty’ (*p* = 0.019) (Figure 7A,B). CAT activity was higher in roots than in leaves in both cultivars (*p* = 0.027). As with CAT activity, in roots, APX and POD activity was significantly enhanced in ‘Bonica’, whereas in leaves the rise was not significant. Increasing the salinity caused a 2-fold increase in APX and POD activity in ‘Bonica’ compared to controls. Overall, APX and POD activity was more enhanced in ‘Bonica’ than in ‘Black Beauty’ (*p* < 0.003). Increasing salt concentration hardly affected APX and POD activity in roots and leaves of ‘Black Beauty’ (Figure 7C–F).

### 2.5. NaCl Induced Changes in Mineral Content

The increase in salinity level boosted the Na accumulation in the leaves and roots compared to the controls in both varieties (Table 2). In ‘Black Beauty’, the Na concentrations in leaves and roots increased significantly as salt stress increased. However, for 160 mM NaCl, there was lower Na accumulation in roots than in leaves (*p* = 0.016). ‘Bonica’ showed a similar response in terms of Na accumulation under saline conditions. A salt concentration of 160 mM NaCl increased leaf Na content by 13-fold in ‘Bonica’ relative to control. Moreover, the rise in salinity level caused ‘Bonica’ to accumulate more Na in roots than in leaves. Furthermore, Na leaf content was higher in ‘Black Beauty’ than in ‘Bonica’. Indeed, the highest salt concentration (160 mM NaCl) led to a 2.7 higher Na content in the leaves of ‘Black Beauty’ than in ‘Bonica’.

Increasing the salinity level contributed to a significant rise in leaf Cl in ‘Bonica’, whereas, in ‘Black Beauty’ a non-significant increase was noted. Yet, at 160 mM NaCl, ‘Black Beauty’ accumulated 1.13-fold the leaf Cl content of ‘Bonica’ (Table 2).

The salt-induced reduction in K was significant in the leaves (*p* = 0.021) and non-significant in the roots (*p* = 0.757) of ‘Black Beauty’. A salt level of 160 mM NaCl, decreased leaf and root K content, respectively, by 41.1% and by 27.9% in ‘Black Beauty’. However, salt stress hardly affected K concentration in leaves and roots of ‘Bonica’ (Table 2). Furthermore, Ca and Mg content were not significantly affected by saline conditions in both cultivars (Table 2).

Under salt stress, leaf P concentration was significantly lowered in ‘Black Beauty’ (*p* = 0.029) and ‘Bonica’ (*p* = 0.052). However, root P content was hardly affected by salinity (Table 2). In addition, no effect on leaf and root S concentrations was found in ‘Black Beauty’ and ‘Bonica’ under saline conditions.

Although the Na/K and Na/Ca ratios in leaves and roots rose by increasing salt concentration in both cultivars compared to their controls, only the Na/K increase was significant in ‘Black Beauty’ (Table 2). In addition, at 160 mM NaCl, the increase in Na/K and Na/Ca ratios in the leaves and roots was more accentuated in ‘Black Beauty’ than in ‘Bonica’ compared to their respective controls. Indeed, in ‘Black Beauty’, the highest salt level increased leaf Na/K and Na/Ca ratios by 12-fold and 15-fold compared to their respective controls, whereas in ‘Bonica’, the same salt level (160 mM NaCl) increased leaf Na/K and Na/Ca ratios, respectively, by 3-fold and 10-fold relative to their respective controls. Moreover, in ‘Black Beauty’, increasing the salt stress to 160 mM NaCl caused roots Na/K and Na/Ca ratios to be elevated by 4-fold and 2-fold relative to their respective controls. On the contrary, in ‘Bonica’, the same salt concentration (160 mM NaCl) caused roots Na/K and Na/Ca ratios to be increased by less than 2-fold relative to controls (Table 2). It is worth noting that the increases in Na/K and Na/Ca ratios under salinity conditions were more accentuated in leaves than in roots.

### 2.6. NaCl Induced Effects on Growth Parameters, Plant Water Status and Development of Leaf Symptoms

**Figure 8:**
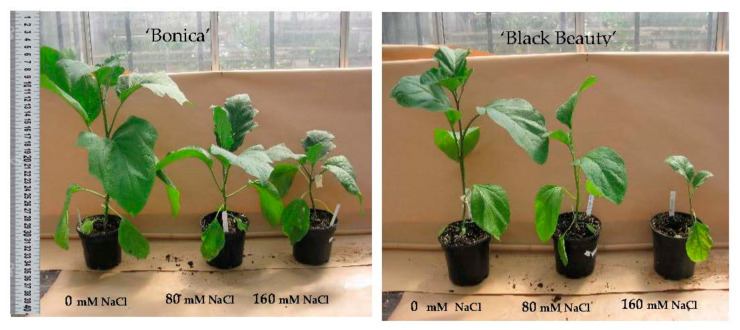
Plant habitus of ‘Bonica’ and ‘Black Beauty’ under different NaCl levels.

To investigate the impact of salinity on biomass we assessed the number of leaves, plant height, aerial FW, and DW (Table 3). Salt stress significantly declined the number of leaves, height, DW, and FW; however, the reduction was stronger in ‘Black Beauty’. A salt concentration of 40 mM NaCl caused a slight increase in the number of leaves and plant height in the two varieties; however, when exposed to higher salt levels, a significant reduction occurred, although only the highest salt concentration significantly diminished the process of leaf initiation in ‘Bonica’ (Table 3; Figure 8). Indeed, a salt concentration of 160 mM NaCl reduced leaf number by 27.3% and 17.6%, respectively, in ‘Black Beauty’ and ‘Bonica’ and plant height by 87.8% and 34.6% in ‘Black Beauty’ and ‘Bonica’ relative to their respective controls (Table 3; Figure 8). The effect of salinity on biomass was more accentuated in ‘Black Beauty’ than in ‘Bonica’. Under saline conditions, the decline of FW and DW was severe in ‘Black Beauty’ (Table 3) and led to reductions in FW and DW of 87.8% and 72.2%, respectively, at 160 mM NaCl. For ‘Bonica’, a more moderate reduction in FW and DW at the 160 mM NaCl level (36.9% and 35.7%) was found.

To investigate the growth and cell expansion, we evaluated the impact of salt stress on TWC. In ‘Black Beauty’, both 80 mM NaCl and 160 mM NaCl significantly reduced the TWC. On the contrary, ‘Bonica’ succeeded in maintaining a stable TWC (Table 3).

**Figure 9:**
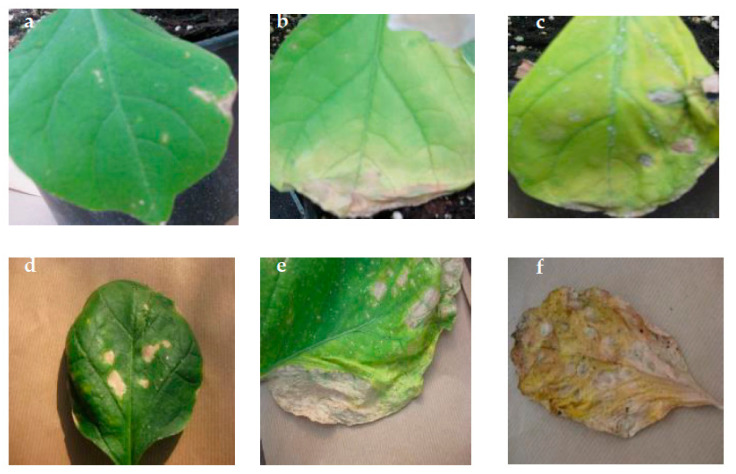
Leaf chlorosis, necrosis and drying in ‘Bonica’ (**a**–**c**) and in ‘Black Beauty’ (**d**–**f**) grown under salt stress.

Under increasing salinity, chlorosis, necrosis and leaf-drying symptoms occurred in both cultivars (Figure 9).

**Figure 10:**
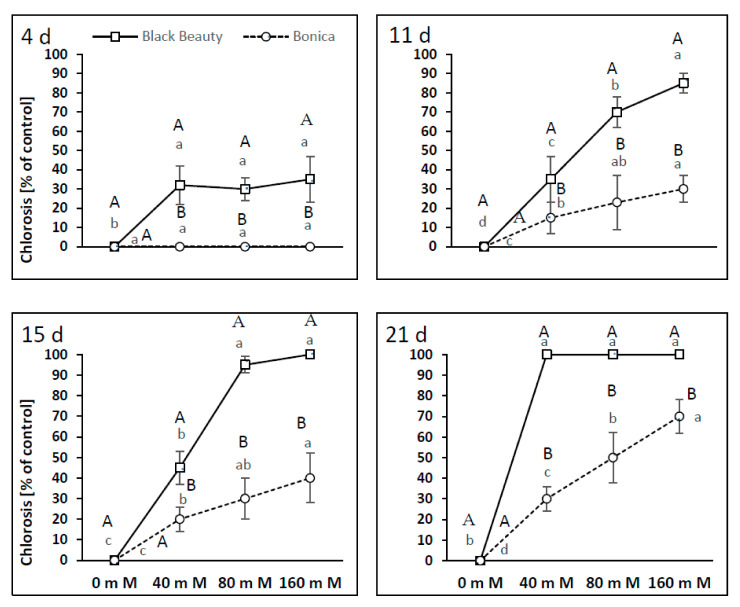
Process of leaf chlorosis evolution and its intensity in percentage of the control after 4, 11, 15 and 21 days of the two eggplant varieties (‘Bonica’ and ‘Black Beauty’) under increasing salinity (0, 40, 80, 160 mM NaCl. Mean value (*n* = 5) ± SE. Significant dissimilarities are shown by different lower-case letters between treatments and by different capital letters between varieties (*p* = 0.05) based on Tukey’s HSD test.

Chlorosis symptoms appeared earlier in ‘Black Beauty’ than in ‘Bonica’ (Figure 10). Four days of NaCl application at 40, 80, and 160 mM generated chlorosis symptoms of about 30% in the leaves of ‘Black Beauty’. After 11 and 15 days, at 80 and 160 mM, chlorosis was aevident in more than 70% of leves (Figure 10). After 21 days under all investigated NaCl concentrations, 100% of the leaves were chlorotic (Figure 10).

The highest salt-induced chlorosis symptoms were noticed at a salt concentration of 160 mM NaCl. The earliest chlorosis symptoms for this concentration were visible on the fourth day (30% of leaves). Eleven and fifteen days later, they could be spotted on more than 70% of the leaves and after 21 days all the leaves were chlorotic. A salt concentration of 40 mM NaCl caused a 30–45% leaf chlorosis after 11 and 15 days, and after 21 days, all the leaves were affected.

In ‘Bonica’, symptoms of chlorosis appeared after 11 days from 40 mM onwards; the chlorotic leaves percentage ranged between 30 and 40% and increased to 70% after 21 days for 160 mM NaCl (Figure 10).

**Figure 11:**
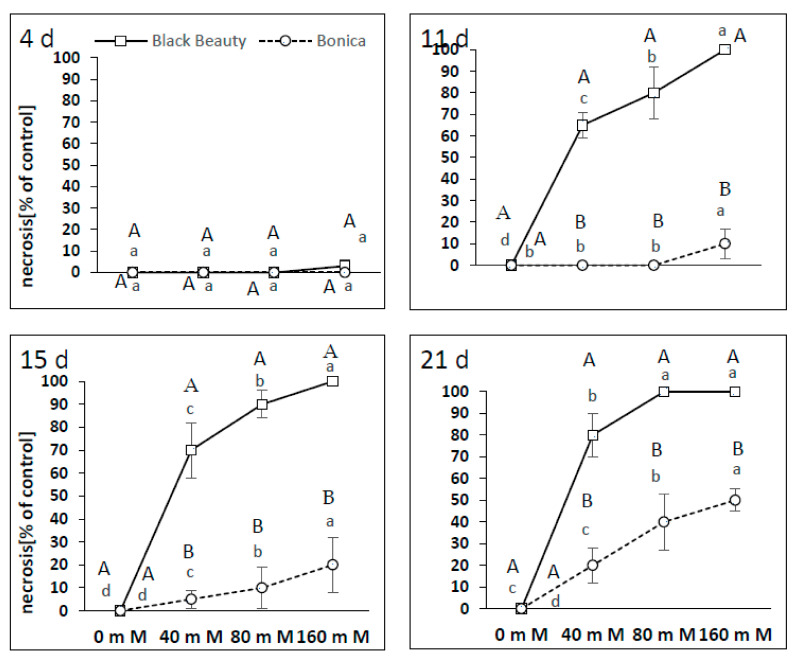
Process of leaf necrosis evolution and its intensity as a percentage of the control after 4, 11, 15, and 21 days of the two eggplant varieties (‘Bonica’ and ‘Black Beauty’) under increasing salinity (0, 40, 80, 160 mM NaCl). Mean value (*n* = 5) ± SE. Significant dissimilarities are shown by different lower-case letters between treatments and by different capital letters between varieties (*p* = 0.05) based on Tukey’s HSD test.

Eleven days after the application of salt stress, all NaCl concentrations caused the appearance of 65 to 100% of necrotic leaves in ‘Black Beauty’ (Figure 11). The leaf necrosis rates for ‘Black Beauty’ at 40, 80, and 160 mM NaCl ranged from 70 to 100% and from 80 to 100% after 15 and 21 days, respectively. In ‘Bonica’, the first necroses (10% of leaves) were found at 160 mM NaCl after 11 days. All salt concentrations (40, 80, and 160 mM NaCl) engendered slight necrosis (5 to 20% of leaves) in ‘Bonica’ after 15 days. Salt concentrations of 80 and 160 mM NaCl induced the clear necrosis of leaf tissues (40 to 50%) after 21 days of the experiment (Figure 11).

**Figure 12:**
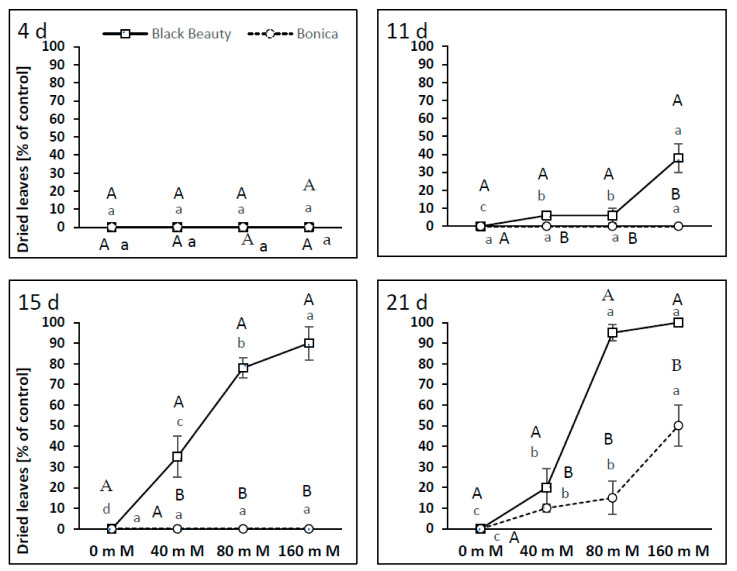
Process of leaf drying and its intensity in percentage of the control after 4, 11, 15, and 21 days of the two eggplant varieties (‘Bonica’ and ‘Black Beauty’) under increasing salinity (0, 40, 80, 160 mM NaCl). Mean value (*n* = 5) ± SE. Significant dissimilarities are shown by different lower-case letters between treatments and by different capital letters between varieties (*p* = 0.05) based on Tukey’s HSD test.

In ‘Black Beauty’, the first symptoms of leaf drying (40% of leaves) were observed after 10 days of plant treatment with 160 mM NaCl (Figure 12). After 15 days of the experiment, leaf drying could be spotted for 78 and 90% of leaves, at 80 and 160 mM NaCl, respectively (Figure 12). Complete leaf drying was noticed in ‘Black Beauty’ after 21 days at 80 and 160 mM NaCl. However, in ‘Bonica’, the first visible symptoms of leaf drying were only observed after 21 days at 160 mM NaCl (50% of leaves) (Figure 12).

Sodium chloride was more toxic in ‘Black Beauty’ than ‘Bonica’, as after 15 days most leaves that were exposed to NaCl were completely wilted and dried.

### 2.7. ACC Deaminase Producing Bacteria Assay

#### 2.7.1. Quantitative Estimation of ACC Deaminase Activity

**Figure 13:**
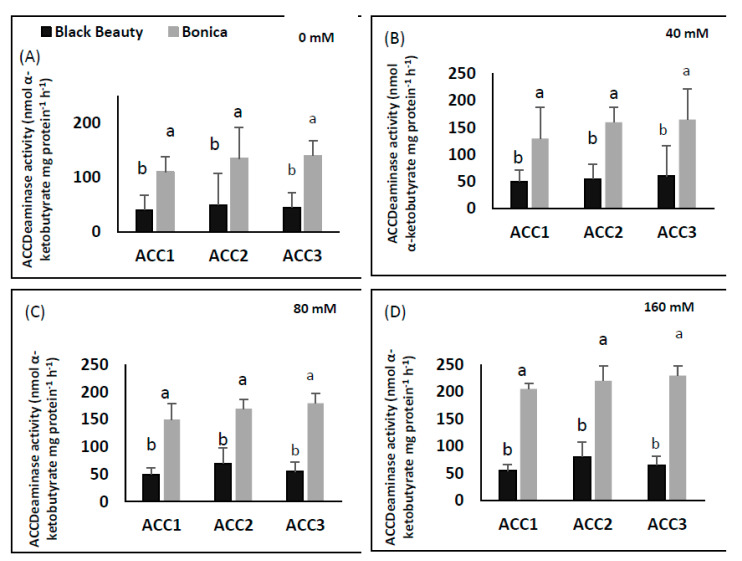
Quantification of ACC deaminase activity (nmol α-ketobutyrate mg protein^−1^ h^−1^) in response to NaCl treatments of four isolates from ‘Black Beauty’ and ‘Bonica’ roots. 0 mM NaCl (**A**), 40 mM NaCl (**B**), 80 mM NaCl (**C**), and 160 mM NaCl (**D**). Values are means of five replicates ± SE (*n* = 5). Significant dissimilarities between varieties (*p* < 0.05) based on Tukey’s HSD test are shown by different lower-case letters.

The ACC deaminase activity of the tested isolates exhibited a variation ranging from 40 to 230 nmol α-ketobutyrate per mg of cellular protein per hour (Figure 13A–D). ‘Bonica’ showed higher ACC deaminase activity in all bacterial strains than ‘Black Beauty’ in all salinity treatments (*p* = 0.016) (Figure 13A–D).

#### 2.7.2. Quantification of Produced Indole Acetic Acid

**Figure 14:**
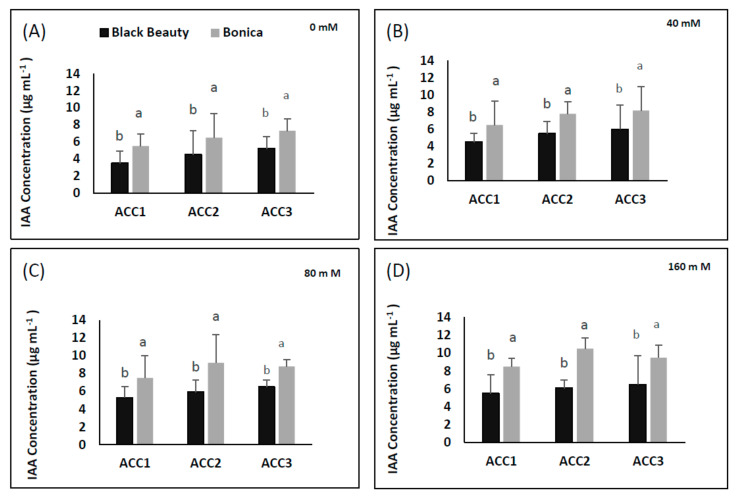
IAA production (nmol α-ketobutyrate mg protein^−1^ h^−1^) in response to NaCl treatments of four isolates from ‘Black Beauty’ and ‘Bonica’ roots. 0 mM NaCl (**A**), 40 mM NaCl (**B**), 80 mM NaCl (**C**), and 160 mM NaCl (**D**). Values are means of five replicates ± SE (*n* = 5). Significant dissimilarities between varieties (*p* < 0.05) based on Tukey’s HSD test are shown by different lower-case letters.

The IAA production of the three ACC deaminase-producing isolates is shown in Figure 14. The accumulation of IAA was significantly more enhanced in ‘Bonica’ than in ‘Black Beauty’ in all treatments for all isolates (*p* = 0.023).

### 2.8. Principal Component Analysis of Morphological and Physiological Responses to Salt Stress

**Figure 15:**
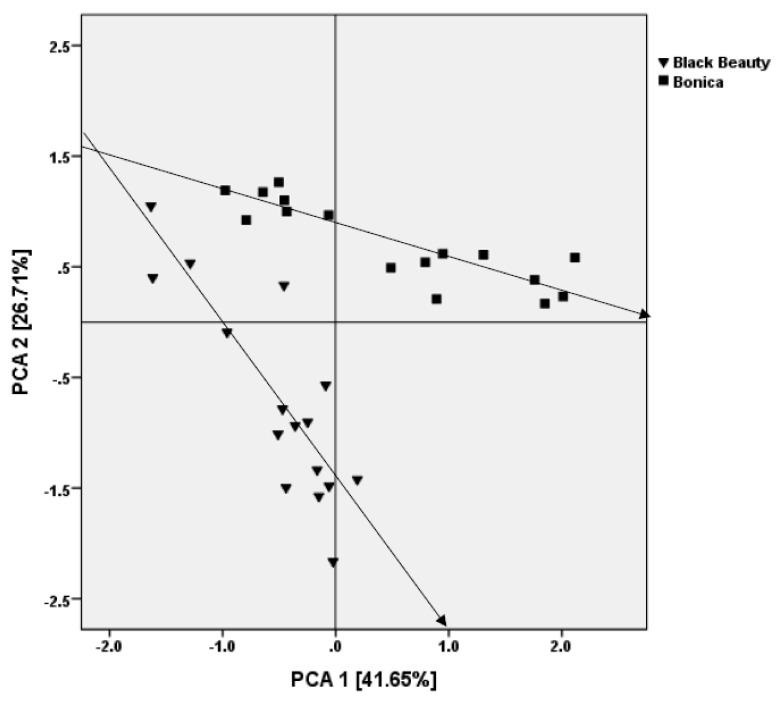
Principal component analysis (PCA) of FW, DW, TWC, ψπ, ψ_H2O_, total phenols, starch, Na/K ratio in leaves, Na/K ratio in roots, EL, POD-L, APX-L, CAT-L, POD-R, APX-R, and CAT-R of the eggplant cultivars grown for 21 days under saline stress. PCA1 is positively correlated with FW, DW, TWC and POD-R, APX-R, and CAT-R and negatively correlated with EL, total phenolic compounds, and starch. PCA2 is positively correlated with ψ_π_ and ψ_H2O_, and negatively correlated with Na/K in leaves, Na/K in roots, and CAT-R. Each data point represents the mean of four replicates. Arrows indicate the increasing salt stress level (▼: ‘Black Beauty’; ■ ‘Bonica’).

A scores scatter plot of the first two PCAs (explaining 61.5% of the variation) shows a clear distinction between ‘Bonica’ and ‘Black Beauty’ after 21 DSS (Figure 15). The loading that had a positive correlation with PCA1 (41.65%) were FW, DW, POD-L, APX-L, CAT-L, POD-R, APX-R, CAT-R and TWC, and those that had a positive correlation with PCA2 (26.7%) were ψ_π_**,** ψ_H2O_, POD-L, APX-L, CAT-L, POD-R and APX-R. The loading of EL, total phenolic compounds, and starch had a negative correlation with PCA1. The loading of Na/K in leaves, Na/K in roots, and CAT-R had a negative correlation with PCA2. For the two varieties, the scores of the PCA moved to lower FW, DW, TWC, ψ_π_ and ψ_H2O_ and higher EL, total phenolic compounds, starch, Na/K ratio in leaves, Na/K ratio in roots, POD-R, APX-R, and CAT-R values under an increasing NaCl concentration. In ‘Black Beauty’, control and salt-stressed plants were highly separated along PCA1 in comparison to ‘Bonica’ (Figure 15). Control and salt-stressed plants showed a clear separation along PCA2 in ‘Black Beauty’.

## 3. Discussion

Previously, [43] showed that contrasting responses in terms of salt stress sensitivity were found in eggplant varieties. In the present work, two eggplant varieties (one salt-sensitive and one salt-tolerant) were subjected to increasing NaCl levels. We focused on photosynthetic machinery, shoot and root mineral accumulation patterns, plant growth, and selected biochemical mechanisms involved in salt stress tolerance. Salt stress causes stomata to close, which decreases CO_2_ uptake, affects the functioning of the photosynthetic apparatus [44], and inhibits plant growth. In our study, rising salinity levels led to stomatal closure and reduced the gross assimilation (A_t_ = net photosynthesis + total respiration in the light), net assimilation and transpiration rate in both eggplant varieties.

In many plants under salt stress, a close relationship has been observed between low leaf water potentials (ψ_H2O_) and both the stomatal closure and non-stomatal decrease in photosynthetic machinery [7,45,46]. However, only ‘Black Beauty’ reduced its leaf water potential to levels below −2.5 MPa, while no correlation between leaf water potential and stomatal conductance was observed in ‘Bonica’. This reveals a tendency for isohydric behavior in ‘Bonica’ where a relative constant midday leaf water potential (ψ_H2O_) is observed, while the control of the plant water balance under increasing salt stress acts through reduced stomatal conductance (gs).

In C3 plants, Rubisco catalyzes the fixation of CO_2_ but also the oxygenation of Ribulose-1,5-BiPhosphate. The fraction of the electron flow dedicated to oxygenation is designated by the ratio Jo/J_t_. Increasing salinity levels do not affect these electron flows in both eggplant cultivars (Figure 2E,F), which varied from 30% to 40% for the oxygenation of RuBP [47,48]. In previous work, [49] showed that, in absence of stress, less than 15% of A_n_ was performed by R_l_. Other researchers stated different percentages while dealing with the fraction of R_l_ relative to A_n_. Indeed, this value ranged from 30–40% in *Nicotiana tabacum* [50], 35 to 40% for *Helianthus annuus* [51], and 50% to more in *Triticum aestivum* [52] under saturating irradiance at temperatures of 20 °C. Our findings suggested that in the absence of salt stress, R_l_ represents 57% and 36% of A_n_, respectively, in ‘Bonica’ and ‘Black Beauty’ (Figure 1). Our results are in agreement with previous work when we consider the temperature range (30–35 °C) during the day in our experiment. Increasing salt concentration to 160 mM NaCl contributed to a substantial rise in this ratio, which reached 79% in ‘Black Beauty’ (40% of the total assimilation rate) and 204% in ‘Bonica’ (32% of the total assimilation rate) after 21 days of salt stress. It is obvious that under salinity conditions, photorespiration represents an important electron sink for the tolerant cultivar ‘Bonica’. Additionally, several reports stated a salt ionic impact on the photosynthetic apparatus for example tomato [53], bell pepper [54] and pea plants [55]. Furthermore, several studies reported a salt ionic impact on photosynthetic apparatus, for example, tomato [53], bell pepper [54], and pea plants [55]. The deterioration of the photosynthetic machinery might be due to the direct impact of Na and Cl or the decline in K. Salt stress caused lower foliar K levels in ‘Black Beauty’ (Table 2), which impacts chloroplast integrity and reduces Rubisco carboxylation activity [56]. Moreover, salt ionic stress induces oxidative stress which may also contribute to the decline in chlorophylls [57,58,59]. Our results showed that ‘Black Beauty’ was sensitive to chlorophyll loss at 80 and 160 mM NaCl, while effects on pigments in ‘Bonica’ were limited. Aside from the decline of the pigment content, salt stress contributes to the aggregation of chloroplasts. Consequently, assimilating organs undergo ultrastructural changes [60]: thylakoid membranes are dilated and mesophyll cells are enlarged [61,62].

Boosting up salinity concentration does not affect mitochondrial respiration (day + night) (Figure 1C–F). ‘Bonica’ and ‘Black Beauty’ succeeded in keeping the same respiration efficiency under salt stress as the control plants. While reviewing the impact of salinity on mitochondrial respiration, [63] emphasized that, although there was high variability in the respiratory behaviors amongst the studied species, susceptible species tended to increase respiratory rates. Indeed, by considering the ratio of R_d_/A_t_, we realized that under increasing salinity levels, respiration acquired a higher fraction of the total assimilation (Figure 2A,B). High respiration rates are closely related to high ATP production [64,65]. It is obvious that under increasing salt concentrations, ‘Bonica’ and ‘Black Beauty’ succeeded in maintaining adequate energy levels. During cellular respiration, organic compounds are oxidized to generate usable chemical energy in the form of ATP [66]. However, this energy might be utilized for several aims. Potentially, ‘Black Beauty’ exploits this energy to achieve osmotic adjustment, while ‘Bonica’ uses it to attain sodium exclusion and tissue tolerance.

Salt stress results in high amounts of reactive oxygen species that contribute to oxidative damage and cause membrane lipid peroxidation, thus decreasing membrane fluidity and selectivity [67,68]. Membranes are most sensitive to stress-induced impairment, and the level of impairment represents a good indicator for salt tolerance [69,70,71,72]. Previous work [73] showed that ’Bonica’ succeeds in maintaining low lipid peroxidation levels under salt stress [69,73,74].

In the present study, the electrolyte leakage (EL) of eggplant leaf disks increased when salt stress intensified (Figure 5B), especially in ‘Black Beauty’, confirming the higher lipid peroxidation of the membranes for this cultivar under salt stress [75], which might be due to higher ROS species and cytotoxic Na levels, since high NaCl levels are closely associated with high lipid peroxidation and membrane damage [76,77,78].

Plants must maintain redox homeostasis and have evolved mechanisms to protect against excessive ROS [79,80,81]. NaCl stress caused the upregulation of the antioxidant enzymes activities, especially in ‘Bonica’. High H_2_O_2_ levels in the cell are efficiently reduced by high activities of CAT and peroxidases such as APX and POD. CAT scavenges the strong oxidant H_2_O_2_ in peroxisomes and converts H_2_O_2_ to water and molecular oxygen [82,83]. In ’Bonica’, salinity impact and CAT activity increased proportionally, contributing to more enhanced protection against ROS compared to the salt-sensitive ‘Black Beauty’. In accordance with our findings, several salt-tolerant varieties showed higher CAT activity, for example tomato, sesame, maize, potato, melon, and wheat [84,85,86,87]. Many studies reported that salt stress enhanced APX activity in salt-tolerant varieties [88]. The relative tolerance of ‘Bonica’ to salt stress might also be explained by the higher APX activity in the leaves with increasing salinity levels. This was also reported in potato, bread, and durum wheat cultivars [85,87,89]. As emphasized by [90,91], APX is crucial for maintaining a normal intracellular level of H_2_O_2_ in plants. Additionally, other peroxidases such as POD activities were higher with increasing salinity levels in the salt-tolerant cultivar, ‘Bonica’. Additionally, in other crops, POD enzymes were involved in salt tolerance such as melon [92], green bean [69], soybean [93], and wheat [87]. POD, which is active intracellularly and extracellularly is an efficient enzyme in the suppression of H_2_O_2_ [94]. The higher salt-tolerant behavior of ‘Bonica’ is supported by its higher ability of enzymatic scavenging of ROS compared to the more salt-susceptible ‘Black Beauty’. Our results suggest that antioxidant enzymes can play a pivotal role in scavenging H_2_O_2_ in tolerant eggplant cultivars. This is in agreement with the scavenging mechanism in other salt-tolerant crops such as tomatoes, sugar beet, potato, and *Plantago* [79,80,85,91].

Phenolic compounds are also strong antioxidants [95,96,97]. However, they hardly contributed to scavenging oxidative stress in our experiment. ‘Bonica’ could maintain its basal levels under increasing salt stress, but in ‘Black Beauty’, biosynthesis decreased with increasing salt stress.

Osmoregulation is one of the key factors of the adaptive mechanisms involved in salt tolerance. The maintaining of the vacuolar ionic equilibrium is ensured by the accumulation of compatible solutes, including amino acids, sugars, and/or other composites [34,98,99].

The rise in carbohydrate accumulation as a mechanism of osmotic regulation in plants subjected to salt stress was reported by several authors [68,100,101]. These osmolytes offer the plant the capacity to alleviate dehydration by ameliorating its potential to maintain the osmotic equilibrium at the cellular level [102]. Other roles attributed to carbohydrates have been reported, such as the buffering of the cellular redox potential, and a source of sufficient energy under severe stress [98]. In the present work, increasing salinity levels caused the susceptible variety, ‘Black Beauty’, to increase its sucrose, glucose, and fructose levels, while the tolerant cultivar ‘Bonica’ maintained or even reduced its sucrose, glucose, and fructose levels with the imposition of salt stress. These results are in accordance with previous findings reported by [103] and [104] in rice and [105] in tomatoes. Overall, the osmoregulation in plants through the enhanced process of compatible solutes accumulation contributes efficiently to maintaining water consumption and cell turgor, which is crucially needed for the regulation of metabolism and cell expansion [106,107]. The susceptible variety (‘Black Beauty’) showed an evident reduction in water potential (ψ_H2O_) and osmotic potential l (ψ_π_). In contrast, the tolerant variety ‘Bonica’ succeeded in preserving more steadiness in terms of ψ_H2O_ and ψ_π_ under saline conditions; this was also observed in previous work [75].

The accumulation of soluble sugars in leaves, as observed in ‘Black Beauty’, might engender a downregulation in carbon metabolism causing a net reduction in CO_2_ uptake [108,109]. In addition, higher sugar levels might contribute to the impairment of the Rubisco expression [110,111].

Moreover, the considerable amount of starch recorded in ‘Bonica’ could potentially generate a mechanical impairment induced by the voluminous starch stack in the chloroplasts, thus causing a negative feed-back on the photosynthetic apparatus [112]. No matter how carbohydrate dynamics changed (sugar or starch accumulation) under increasing salt stress, both varieties showed a similar photosynthetic response (A_n_ and A_t_) (*p* > 0.05).

As described by [40], after reaching toxic salt levels in the old leaves (which were unable to grow and lost their capacity to cope with salt influx as they used to do), the ion-specific step of the salt stress began. The salt stress imposition increased Na and Cl levels in the leaves and roots of both cultivars. The high leaf Na^+^ content increase in the sensitive cultivar ‘Black Beauty’ was strongly linked to a more reduced osmotic potential (ψ_π_) when compared to the tolerant cultivar ‘Bonica’.

High competition between the consumed NaCl and other minerals, particularly K, contributes to the lack of K [113]. However, a decline in K content was only found in ’Black Beauty’ at higher salt levels. Consequently, Na/K and Na/Ca ratios were lower in ‘Bonica’ than in ‘Black Beauty’. The salt susceptibility and tolerance in plants is closely linked to these ionic ratios. Indeed, tolerant cultivars are characterized by lower Na/K and Na/Ca ratios [114,115]. To achieve full growth in a normal way, especially at the cellular level, a low cytosolic Na/K ratio is crucially needed by plants. Under increasing salinity, K specific transporters could be inhibited by Na during the competition with K uptake. Consequently, a toxic level of Na occurs in parallel with a lack of K content for osmotic regulation and enzyme stability [115,116]. To cope with salt stress, plants developed several adaptive mechanisms such as the modulation of cell Na consumption and long-distance Na transport [117]. During the subjection to salt stress, some species show a tendency for root Na accumulation and shoot Na exclusion. Such plants are known as Na excluders. Other species tend to enhance Na accumulation in the shoots and are referred to as Na includers [26]. ‘Bonica’ accumulated two times more Na in roots than in shoots, likely revealing the presence of a mechanism of controlled long-distance transport from roots to shoots, whereas at the highest salt concentration, the Na accumulation in shoots was higher in ‘Black Beauty’ than in ‘Bonica’. The success of ‘Bonica’ in keeping lower leaf Na content than ‘Black Beauty’ proves that its control of the long-distance process is more efficient than the one in ‘Black Beauty’. In monocotyledonous plants, the leaf sodium exclusion system occurs as the major mechanism involved in salt tolerance. As emphasized by [68,118], the limited Na consumption of roots and the reduced rate of transport in the xylem from root to shoot contributes to the so-called Na exclusion. According to [68], the achievement of the Na exclusion mechanism is ensured by a limited charging in the xylem, or effective Na suppression from the upper part of the root system and the base of the shoot. However, to cope with the considerable leaf Na accumulation, several glycophytes have the tendency to exclude Na and Cl^−^ from the cytosol via vacuole compartmentalization [119,120,121]. We did not study this mechanism in eggplant; however, the Na exclusion mechanism was likely to be more efficient in ‘Bonica’ than in ‘Black Beauty’.

The impact of salinity on plant morphology and growth parameters, such as shoot and roots, leaf area, fresh and dry weight, plant height, yield, and yield quality traits, is widely reported [122,123,124]. The impact on the morphological traits of eggplant cultivars was similar to those observed in previous work [75]. Salt stress substantially decreased leaf initiation, plant height, and fresh and dry weight in the salt-susceptible variety, ‘Black Beauty’, and moderately affected the same parameters in the salt-tolerant cultivar, ‘Bonica’. In addition to that, chlorosis and necrosis symptoms started earlier and to a higher extent in ‘Black Beauty’. High NaCl content in the root environment limits water availability, thus contributing to a physiological drought [125] that led to a reduction in leaf tissue water content and fast leaf senescence in ‘Black Beauty’ after 15 days of salt stress under 80 and 160 mM NaCl. The different development rates of senescent leaves explain the difference in TWC measured at the whole-plant level between the cultivars. Higher salt treatments impacted TWC strongly in ‘Black Beauty’, while ‘Bonica’ succeeded in preserving its TWC up to 160 mM NaCl, as already reported in previous work [94]. According to [44], in most species, the threshold for keeping a steady TWC is about 40 mM NaCl. So far, in accordance with salt stress assays (soil or soilless experiments) and plant behavior (halophyte or glycophyte), thresholds range from 200 mM NaCl in mangrove (*Brugiera parviflora*) [3], 200 mM NaCl in jute species (*Corchorus olitorius*) [126], and 90 mM in *Atriplex griffithii* [127] to 40 mM in okra (*Abelmoschus esculentus*) [128].

ACC deaminase activity exhibited by PGPR was proven to play a key role in improving growth and stress tolerance in plants under stressed conditions [129,130]. ACC deaminase, as a microbial enzyme, contributes to dissociating stress-induced ACC into ammonia and α-ketobutyrate, which produces ethylene that causes serious damage to the physiology, growth and development of plants [131]. In the present work, the salt tolerance response of ‘Bonica’ was associated with higher ACC deaminase activity in all bacterial strains, specifically in ‘ACC2′ and ‘ACC3′ when compared to ‘Black Beauty’, which showed lower values. The results of the current study agree with previous published works, reporting that bacteria provided by ACC deaminase activity can counteract the toxic impact of salt stress [132,133]. The most intensive enzymatic activity of ACC deaminase produced by ‘ACC2′ and ‘ACC3′, i.e., the conversion of nitrogen source ACC into α-ketobutyrate, was confirmed according to [129,131].

Under salt stress, PGPB contribute to enhancing additional IAA production, which may be useful in stimulating root growth, counteracting the inhibition impact of salinity on roots and shoot growth, and ameliorating plant physiological features [134].

In our study, ‘Bonica’ exhibited higher IAA accumulation in all isolates, specifically in ‘ACC2′ and ‘ACC3′ compared to ‘Black Beauty’ in response to increasing salinity, which might be explained by their better capacity to tolerate salt stress. This is consistent with previous work reported by [135,136].

## 4. Materials and Methods

### 4.1. Plant Materials and Treatments

Seeds of salt-tolerant (‘Bonica’) and salt-susceptible (‘Black Beauty’) eggplant cultivars (*Solanum melongena* L.) were used. Germination of eggplant was performed in trays filled with peat and wetted with distilled water. Trays were placed in a controlled growth chamber (BioevoPeak) under 150 µmol m^−2^ s^−1^ of photon flux density (provided by cool-white fluorescent lamps), at a constant temperature of 25 °C and 70% of relative humidity (RH). Twenty-five-day-old seedlings showing their second true leaf were chosen, transferred to 2 L plastic pots (17 × 13 cm) filled with peat and maintained in a heated glasshouse with a minimum temperature setpoint of 21 °C (located at 51°02′ N, 03°42′ E) at the Department of Plants and Crops, Ghent University, Belgium. The temperature ranged between 22 °C and 27 °C while the daily maximum photon flux density averaged 340 μmol m^−^^2^ s^−^^1^. A solution of full-strength Hoagland (250 mL) [137] was used to irrigate each seedling twice a week, starting 36 days after the transfer to the greenhouse (4th leaf stage). The tolerant and susceptible seedlings were randomly divided into 4 groups as follows: 0 (control), 40, 80, and 160 mM NaCl. We irrigated control plants twice a week with 250 mL of distilled water for 30 days, whereas salinity-stressed plants were irrigated twice a week with 250 mL of 40, 80, and 160 mM NaCl solution for 30 days. Irrigation frequency and dose per plant remained unchanged. Twenty plants per cultivar (5 plants/block) were subjected to each treatment. A randomized complete block design was adopted in our experiment with four replications for each treatment and cultivar.

### 4.2. Gas Exchange Measurements

To investigate the impact of salinity on gas exchange parameters, measurements were conducted once a day (between 09:00 h and 12:00 h) on four randomly selected plants from each of the four treatments. This was conducted after 13 days of salt stress (DSS) (13 June 2012) and 21 DSS (21 June 2012). Measurements were performed on sunny days. Gas exchange measurements were conducted with a portable photosynthesis system (model LI-6400; Li-Cor Biosciences, Lincoln, NE, USA) fitted with a fluorescence head (6400–40 Leaf Chamber Fluorometer, Li-Cor Biosciences, Lincoln, NE, USA). All measurements were conducted on the youngest fully developed leaves. We determined light-saturated net photosynthesis (A_n_, µmol CO_2_ m^−2^ s^−1^), stomatal conductance (g_s_, mmol H_2_O m^−2^ s^−1^), and transpiration rates (E, mmol H_2_O m^−2^s^−1^) in the chosen plants. To measure the traditional dark respiration by night, it was necessary to cover leaves in the early morning (R_n_, µmol m^−2^ s^−1^). According to [33], we calculated the mitochondrial respiration by day (R_d_, µmol m^−2^s^−1^) from R_n_. R_d_ was extrapolated at different temperatures using a Q_10_ relation [138] as follows:

R_d_ = R_n_ Q_10_ ^(T_d_ − T_n_)/10^ (Q_10_ = 2.2) where T_n_ is the leaf temperature at which R_n_ was measured, and T_d_ is the leaf temperature at which R_d_ was calculated. For Mediterranean climates, Q_10_ is expected to be around 2.2 [138].

The chamber temperature of the fluorescence head was adjusted to fit the actual temperature assayed in the treatment environment at the beginning of the measurement (25 °C). Under treatment conditions, the light source of the fluorescent head was kept at 1500 µmol m^−2^s^−1^, and CO_2_ was kept at 400 µmol CO_2_ mol^−1^.

The maximum quantum yield of PSII (F_v_/F_m_) was measured on dark-adapted leaves (30 min). Red actinic light (1500 µmol m^−2^s^−1^) was then switched on, and the quantum yield of PSII electron transport (Φ_PSII_) and efficiency of energy capture by the open PSII reaction center were determined by measuring steady-state fluorescence and maximum fluorescence during 0.5 s of a light saturation pulse of 7000 µmol m^−2^s^−1^, recorded on the adaxial surface of the same leaves used for gas exchange measurements. Φ_PSII_ was calculated as follows:

Φ_PSII_ = (F_m_’ − F_s_)/F_m_’

where F_m_ = maximum fluorescence in leaves acclimated to darkness, F_m_’ is the maximum fluorescence in leaves submitted to ambient light, and F_s_ is the steady fluorescence in leaves acclimated to ambient light.

The total electron flow (J_t_) can be obtained from the quantum yield of PSII (Φ_PSII_), the light intensity incident on the leaf (PAR), the fractional absorption of light by the leaf (a), and the absorptance of PSI + PSII (f) as follows [139]:

J_t_ = Φ_PSII_ × PAR × a × f (µmol m^−2^ s^−1^)

where ‘a’ equals 0.84, ‘f’ equals 0.5 [140] and PAR = 1500 µmol m^−2^ s^−1^.

According to [141], the partitioning of electrons between photosynthesis (J_c_) and photorespiration (J_o_) can be calculated using the values of the electron transport rate (J_t_), A_n,_ and mitochondrial respiration rate in the light (R_d_):

J_c_ = 1/3 [J_t_ + 8(A_n_ + R_d_)] and J_o_ = 2/3 [J_t_ − 4(A_n_ + R_d_)] (µmol m^−2^ s^−1^)

Photorespiration (R_l_, µmol CO_2_ m^−2^ s^−1^), was measured as follows [33]:

R_l_ = 1/12 [J_t_ − 4(A_n_ + R_d_)] (µmol m^−2^ s^−1^)

Total assimilation rate was determined as A_t_ = (A_n_+ R_d_+ R_l_) (µmol m^−2^ s^−1^). R_d_/A_t_ is utilized to quantify the fraction of the total assimilation that goes to respiration under elevated saline stress.

### 4.3. Plant Growth, Plant Water Status, and Development of Leaf Symptoms

After exposition to salt stress for 30 days (30 DSS), we randomly selected eight plants for each treatment. We determined the fresh weight (FW) of shoots and leaves, and we counted the number of leaves. The samples were then oven-dried at 70 °C for 48 h to determine the dry weight (DW). The tissue water content (TWC) was calculated using the formula: TWC = (FW − DW/FW). The Scholander pressure chamber (model 1000, PMS Instrument Company, Albany, OR, USA) was used to determine the leaf water potential (ψ_H2O_) of the youngest fully expanded leaves. We determined the leaf osmotic potential (ψ_S_) according to [142] following the osmometry of leaf sap expressed using a press method. Measurements were performed in four repetitions.

The evolution of leaf chlorosis, necrosis, and drying in both cultivars was assessed on four dates (4 DSS, 11 DSS, 15 DSS, and 21 DSS) and in five replicates per experimental plot. Leaves showing symptoms (treated by 0, 40, 80, and 160 m M NaCl) were counted and expressed as a percentage of the control (treated by water free of NaCl).

### 4.4. Metabolites Extraction and Analysis

After 30 days of salt stress, we sampled fully developed upper leaves (2 leaves/replicate in a bulked sample) between 12 h and 14 h from four plants, in each treatment (1 plant/block), and for each variety. We ground the leaf material in liquid nitrogen and stored it at −80 °C until analysis.

The pigments were extracted from 0.15 g fresh weight using 80% acetone (***v***/***v***). The mixture was kept in the dark at room temperature until the leaf tissue was completely bleached. After complete extraction, the extract was centrifuged for 5 min at 5000× *g* and then brought up to a final volume of 15 mL using 80% acetone. The absorbance of the extracts was read spectrophotometrically at 645 and 663 nm using a spectrophotometer (UV-2550, Shimadzu, Japan). Finally, the concentrations of each pigment in µg g-1 fresh weight (FW) were calculated according to the method previously described by [143,144].

Soluble sugars were extracted from 20 mg fresh weight, by 1 mL of ethanol (80%) at 70 °C for 10 min and further at 45 °C for 3 h, followed by centrifugation at 5000× *g* for 5 min [75]. HPLC was used to determine the sugars (Waters; CarboPac MA1 column with companion guard column, eluent: 50 mM NaOH, 22 °C).

The rest of the ethanol insoluble material was rinsed twice with 80% ethanol, and the pellet was treated with HCl 1 M for 2 h at 95 °C for starch hydrolysis. Starch extraction and quantification were carried out according to [75] and based on the enzymatic reduction in NADP^+^.

### 4.5. Enzymatic Assays

For protein and enzyme extractions, 0.5 g of leaf and root samples were homogenized with 50 mM potassium phosphate buffer (pH 7.8) containing 1 mM EDTA-2Na and 7% (*w/v*) polyvinylpolypyrrolidone (PVPP). The whole extraction procedure was carried out at 4 °C. The homogenates were centrifuged at 4 °C for 15 min at 13,000× *g*, and enzyme activity was measured using the supernatants. Protein was quantified as described by [145], utilizing bovine serum albumin as a standard.

According to [146], we assayed catalase (CAT) activity (EC 1.11.1.6) by determination of the level of decomposition of H_2_O_2_ (ε = 2.3 mM^−1^ cm^−1^) at 240 nm. This activity was measured in a reaction mixture containing 1900 µL of potassium phosphate buffer (50 mM, pH 7.0 not containing EDTA), 100 µL sample and 1000 µL H_2_O_2_ (30 mM). CAT activity was expressed as µmol H_2_O_2_ decomposed min^−1^ mg^−1^ proteins.

Ascorbate peroxidase (APX) activity (EC 1.11.1.11) was determined according to [147]. The reaction mixture contained 50 mM of potassium phosphate buffer (pH 7.0), 4.4 µL ascorbate (1 mM) and 10 µL EDTA-2Na (0.5 M). Adding H_2_O_2_ started the reaction, and ascorbate oxidation was determined at 290 nm for 1 min. Activity was quantified using the extinction coefficient, e = 2.8 mM^−1^ cm^−1^. Each sample was measured in three repetitions. Results were expressed as µmol oxidized ascorbate min^−1^ mg^−1^ proteins.

According to [148], we quantified the guaiacol peroxidase (POD) activity (EC 1.11.1.7). The reaction solution includes 100 µL of plant extract supplemented by 700 µL of 0.05 M phosphate buffer (pH 7.8) and 200 µL of guaiacol (25 mM). The reaction began by adding 100 µL of H_2_O_2_. The absorbance elevation generated by oxidation of guaiacol to tetra guaiacol was recorded for 3 min at 436 nm. POD activity was estimated from the extinction coefficient, ε = 25.5 mM^−1^ cm^−1^. Results were expressed as µmol oxidized guaiacol min^−1^ mg^−1^ proteins.

### 4.6. Mineral Content

Leaves and roots samples were harvested from four plants in every treatment (1 plant/block) and for every cultivar, washed, oven-dried at 70 °C for 48 h, and finally, ground. The determination of P, K, Ca, Mg, S, and Na contents was performed by ICP-OES after dry-ashing at 550 °C. A potentiometric analysis using an ion-selective electrode (VWR, Leuven, Belgium) for chlorides was used.

### 4.7. Electrolyte Leakage (EL)

Electrolyte leakage (EL) was measured according to [149]. Briefly, three leaf disks (2 mm in diameter) were cut after 30 days of salt stress (30 DSS) from three selected and fully washed expanded leaves. After being placed in a glass vessel containing 10 mL of sterile distilled water, disks were shaken for 5 h, then the initial EC1 was determined via an Electrical Conductivity Meter (Beckman, instrument Inc., Cedar Grove, NJ, USA). Then, we measured the EC2 after autoclaving leaf disks (121 °C for 15 min). Finally, the EL (%) was calculated as follows: EL (%) = (EC1/EC2) × 100.

### 4.8. Total Phenolic Content

Leaf total phenolic content was determined (after 30 DSS) according to [150]. In short, the reaction mixture obtained by adding the methanolic extract (10 μL) to distilled water (490 μL) was supplemented by the Folin–Ciocalteu reagent (500 μL), then shaken (for 6 min) and incubated at room temperature for 2 h. The sample absorbance was recorded at 765 nm versus blank. Gallic acid was used as standard. The total phenolic amount was determined using the calibration curve, and the results were expressed as mg of gallic acid equivalent per g dry weight (mg of GAE/g DW).

### 4.9. ACC Deaminase Producing Bacteria Assay

The enzyme 1-aminociclopropane-1-carboxylase (ACC) deaminase activity is one of the key traits used by PGPB to decrease ethylene levels under salt stress. The ACC, deaminase, converts ACC into ammonia and α-ketobutyrate.

#### 4.9.1. Collection of Rhizospheric Soil Sample

The root samples of ‘Bonica’ and ‘Black Beauty’ were collected from 6 pots for each cultivar in every treatment during May and June 2021 in a heated glasshouse (36°50′ N, 10°11′ E) at the Department of Plant Physiology and Biotechnology, National Institute of Agronomic Research, Tunisia. Four plants/cultivar/treatment were collected from each pot and brought to the laboratory in closed plastic bags for further analysis. The roots were separated from each plant, crushed and mixed together to form one composite pool of root sample.

#### 4.9.2. Isolation of Bacteria and Qualitative Estimation of ACC Deaminase Activity

The isolation of bacteria and the qualitative determination of ACC deaminase activity were carried out according to [151]. Briefly, the bacteria were isolated from the roots sample by serial dilution technique in Luria-Bertani (LB) medium. The morphologically different colonies were subjected to ACC deaminase activity screening on sterile minimal DF (Dworkin and Foster) salts media corrected with 3 mM ACC as the sole nitrogen source [152]. The inoculated plates were incubated at 28 °C for 3 days and growth was observed daily. The growing colonies were considered as ACC deaminase producers and were purified by sub-culturing the isolates.

Fifteen bacterial strains were isolated from roots of ‘Bonica’ and ‘Black Beauty’ on enrichment media, of which, three strains had the capacity of growing on DF minimal salt medium supplemented with 3 mM ACC as a nitrogen source, implying ACC deaminase activity. The ACC deaminase activity of these three isolates, named as ‘ACC1’, ‘ACC2’ and ‘ACC3’, was further quantified in terms of α-ketobutyrate production.

#### 4.9.3. Quantification of ACC Deaminase Activity

The quantitative determination of ACC deaminase activity was performed according to [152]. This method assesses the amount of α-ketobutyrate produced via catalyzation of the deamination reaction of sole nitrogen source, ACC in DF minimal salt broth media at 540 nm. The ACC deaminase activity was expressed in mmol α-ketobutyrate mg protein^−1^ h^−1^.

#### 4.9.4. Indole Acetic Acid Production by Bacterial Isolates

According to [129], the bacterial strains were inoculated in LB medium corrected with 5 mM tryptophan and incubated in an orbital shaker for 7 days at 28 °C at 200 rpm. The IAA quantification was achieved via the colorimetric method using Salkowski reagent (0.5 M FeCl_3_ + 70% perchloric acid). Development of red color (which indicates the formation of indolic compounds) with the addition of Salkowski reagent and cell-free culture supernatant (4:1) was measured by a UV–VIS spectrophotometer at 530 nm [153]. The concentration of IAA can be determined with a standard curve of pure indole-3-acetic acid (IAA, Hi-media) ranging between 0 and 100 mg mL^−1^.

### 4.10. Statistical Analysis

A completely random design was used in performing all analyses. The detection of significant dissimilarities between the treatments and varieties was achieved via the software of SPSS Statistics 25 (IBM SPSS Statistics) after subjecting all collected data to a two-way analysis of variance (ANOVA). Means comparison was made using Tukey’s multiple range test (*p* = 0.05). A Pearson correlation analysis was performed between gas exchange parameters, chlorophyll content, parameters of the plant water balance and leaf Na contents. (*p* = 0.05). We performed the principal component analysis (PCA) on FW, DW, TWC, total chlorophyll content, osmotic potential, leaf water potential, total phenols, starch, Na/K ratio in leaves, Na/K ratio in roots, EL, CAT activity, APX activity and POD activity of both eggplant varieties. PCAs with eigenvalues > 1, thus explaining more than a single parameter alone, were examined. For these principal components, a varimax rotation was applied to the factor loadings. According to [154], this rotation offers simpler factors, relating parameters essentially to one principal component axis.

## 5. Conclusions

Our results indicate that different cellular processes explain the stress sensitivity/tolerance of the eggplant cultivars.

Photorespiration is an alternative electron sink and at higher salt levels, ‘Bonica’ uses this pathway to a greater extent than ‘Black Beauty’. Photosynthesis is strongly affected by increasing salt stress in both cultivars, though mitochondrial respiration remains unaffected in absolute values and the ratio R_d_/A_t_ increases.

Leaf osmoregulation, K and Na leaf levels, and ion homeostasis are important mechanisms conferring salinity tolerance. Osmotic adjustment was strongly present in the susceptible ‘Black Beauty’ and correlated with higher sugar levels under saline conditions. Additionally, g_s_ and ψ_S_ were significantly correlated in ‘Black Beauty’: the more stomata that were closed, the more negative the osmotic potential. The tolerant ‘Bonica’ was more successful to keep Na to the root system compared to the susceptible ‘Black Beauty’. ‘Bonica’ could also better maintain its cellular homeostasis as indicated by lower membrane leakage levels and a more performant enzymatic antioxidative system, which led to a higher biomass production.

Two putative strains, ‘ACC2’ and ‘ACC3’, were found to possess other growth-promoting properties, such as the production of IAA. The efficiency of these strains in reducing salt stress and promoting plant growth was evident in ‘Bonica’.

Finally, the protection of plant tissues against salt stress was achieved by a highly effective antioxidant enzymatic machinery defense, which alleviates the damaging effects of ROS and makes ‘Bonica’ more tolerant.

## Figures and Tables

**Table 1:** Effect of increasing NaCl concentration on chlorophyll a (Chl*a*), chlorophyll b (Chl*b*), Chl*a/b*, total chlorophyll (Chl*a* + *b*) and carotenoids in leaves of the eggplant cultivars after 30 days of salt stress.

cv	NaCl (mM)	Chl*a* (µg g^−1^ FW)	Chl*b* (µg g^−1^ FW)	Chl*a/b*	Chl*a* + *b* (µg g^−1^ FW)	Carotenoids (µg g^−1^ FW)
‘Black Beauty’	0	689.9 ± 1.7 aA	340.6 ± 5.7 aA	2.02 ±5.6 aA	1030.5 ± 3.4 aA	240.9 ± 2.5 aA
	40	758.9 ± 1.5 aA	345.4 ±5.4 aA	2.2 ±4.6 aA	1104.3 ± 4.1 aA	273.6 ± 5.9 aA
	80	326.8 ± 1.4 bB	250.3 ± 5.7 bA	1.3 ±6.9 bB	577.1 ± 2.5 bB	154.9 ± 3.7 bA
	160	153.8 ± 35.2 cB	154.4 ± 4..2 cB	0.9 ± 4.3 cB	308.2 ± 4.6 cB	117.1 ± 2.3 cA
‘Bonica’	0	585.3 ± 2.8 aA	288.4 ± 6.3 aA	2.02 ±8.6 aA	873.7 ± 3.2 aA	147.6 ± 2.8 aB
	40	611.5 ± 5.7aA	298.0 ± 5.4 aA	2.05 ± 6.9 aA	909.5 ± 2.3 aA	140.8 ± 1.9 aB
	80	485.2 ± 6.3aA	239.3 ± 2.6 aA	2.02 ±7.5 aA	724.6 ± 5.2 aA	124.8 ± 1.7 aA
	160	454.5 ± 5.4 aA	248.3 ± 4.8 aA	1.85 ±10.9 aA	702.8 ± 3.6 aA	105.7 ± 1.6 aA

Data are means ± SE of four replicates. Significant dissimilarities are shown by different lower-case letters between treatments and by different capital letters between varieties (*p* = 0.05) based on Tukey’s HSD test.

**Table 2:** Effect of increasing salinity level on K, Ca, Mg, Na, P, S, and Cl contents (based on DW) and on Na/K and Na/Ca ratio in leaves and roots of the eggplant varieties (After 30 days of salt stress).

Cultivar	Tissue	NaCl (mM)	K (g/100 g)	Ca (g/100 g)	Mg (g/100 g)	Na (g/100 g)	P (g/100 g)	S (g/100 g)	Cl (g/100 g)	Na/K	Na/Ca
‘Black	Leaves	0	6.4 ± 0.4 aA	3.1 ± 0.4 aA	0.5 ± 0.01 aA	0.4 ± 0.05 cA	0.7 ± 0.02 aA	0.2 ± 0.06 aA	2.8 ± 0.16 aA	0.1 ± 0.0 bA	0.1 ± 0.03 aA
‘Beauty’		40	6.7 ± 0.4 aA	3.2 ± 0.3 aA	0.6 ± 0.05 aA	0.9 ± 0.08 cA	0.7 ± 0.06 aA	0.2 ± 0.06 aA	2.8 ± 0.16 aA	0.1 ± 0.02 bA	0.2 ± 0.05 aA
		80	5.3 ± 0.4 abA	3.1 ± 0.2 aA	0.5 ± 0.01 aA	1.8 ± 0.11 bA	0.6 ± 0.05 abA	0.2 ± 0.06 aA	4.5 ± 0.69 aA	0.3 ± 0.08 abA	0.7 ± 0.12 aA
		160	3.7 ± 0.3 bB	2.8 ± 0. 2 aA	0.5 ± 0.01 aA	3.8 ± 0.14 aA	0.5 ± 0.05 bA	0.2 ± 0.04 aA	6.9 ± 0.92 aA	1.2 ± 0.12 aA	1.5 ± 0.15 aA
	Roots	0	1.8 ± 0.28 aA	1.3 ± 0.29 aA	0.2 ± 0.04 aA	1.1 ± 0.10 bA	0.5 ± 0.10 aA	0.3 ± 0.03 aA	*	0.4 ± 0.03 bB	0.9 ± 0.03 aA
		40	1.6 ± 0.55 aA	1.2 ± 0.35 aA	0.2 ± 0.07 aA	1.2 ± 0.08 bB	0.4 ± 0.07 aA	0.2 ± 0.04 aA	*	0.8 ± 0.51 abB	0.9 ± 0.48 aA
		80	1.1 ± 0.11 aA	1.3 ± 0.16 aA	0.2 ± 0.03 aA	1.2 ± 0.10 bB	0.4 ± 0.08 aA	0.2 ± 0.05 aA	*	0.9 ± 0.37 abB	0.8 ± 0.30 aB
		160	1.3 ± 0.21 aA	1.1 ± 0.13 aA	0.2 ± 0.01 aA	2.4 ± 0.19 aA	0.4 ± 0.07 aA	0.3 ± 0.03 aA	*	1.7 ± 0.50 aB	2.2 ± 0.39 aA
‘Bonica’	Leaves	0	4.8 ± 0.61 aB	2.8 ± 0.22 aA	0.6 ± 0.06 aA	0.1 ± 0.010 cA	0.7 ± 0.07 aA	0.2 ± 0.08 aA	1.2 ± 0.35 cB	0.1 ± 0.0 aA	0.04 ± 0.0 aB
		40	5.0 ± 0.68 aB	3.3 ± 0.12 aA	0.6 ± 0.05 aA	0.5 ± 0.07 bB	0.6 ± 0.07 abA	0.2± 0.04 aA	3.3 ± 0.31 bcA	0.1 ± 0.02 aA	0.2 ± 0.01 aA
		80	4.6 ± 0.45 aA	3.5 ± 0.35 aA	0.6 ± 0.04 aA	1.3 ± 0.08 aB	0.6 ± 0.04 abA	0.2 ± 0.03 aA	4.7 ± 0.59 abA	0.3 ± 0.11 aA	0.4 ± 0.07 aA
		160	4.9 ± 0.43 aA	3.5 ± 0.35 aA	0.7 ± 0.05 aA	1.4 ± 0.08 aB	0.5 ± 0.08 bA	0.2 ± 0.03 aA	6.1 ± 0.95 aA	0.3 ± 0.05 aB	0.4 ± 0.09 aB
	Roots	0	1.2 ± 0.14 aA	1.4 ± 0.14 aA	0.3 ± 0.06 aA	1.5 ± 0.13 bA	0.7 ± 0.04 aA	0.3 ± 0.07 aA	2.6 ± 0.31 a	1.3 ± 0.03 aA	1.3 ± 0.01 aA
		40	0.8 ± 0.09 aB	1.6 ± 0.12 aA	0.2 ± 0.03 aA	1.8 ± 0.11 bA	0.7 ± 0.02 aA	0.3 ± 0.04 aA	2.8 ± 0.16 a	2.4 ± 0.07 aA	1.3 ± 0.03 aA
		80	1.0 ± 0.25 aA	1.2 ± 0.24 aA	0.3 ± 0.04 aA	1.9 ± 0.13 bA	0.5 ± 0.02 aA	0.2 ± 0.03 aA	2.2 ± 0.29 a	1.7 ± 0.42 aA	1.4 ± 0.20 aA
		160	1.2 ± 0.33 aA	1.3 ± 0.32 aA	0.2 ± 0.03 aA	2.5 ± 0.09 aA	0.7 ± 0.13 aA	0.3 ± 0. 00 aA	6.4 ± 0.78 a	2.2 ± 0.24 aA	2.4 ± 0.24 aA

Values are means ± standard errors of four replicates. *: No data because of the lack of sufficient plant material. Significant dissimilarities are shown by different lower-case letters between treatments and by different capital letters between varieties (*p* = 0.05) based on Tukey’s HSD test.

**Table 3:** Impact of NaCl concentration on morphology and plant water status of two eggplant varieties (after 30 days of salt stress). Plant morphological parameters include leaf number, plant height, fresh weight of plant aerial part (FW), dry weight of plant aerial part (DW), and water status of the plant including tissue water content (TWC), midday leaf water potential (ψ_H2O_) and osmotic leaf potential (ψ_π_).

Cv	NaCl (mM)	Number of leaves	Plant Height (cm)	FW (g)	DW (g)	TWC (g/g)	ψ_H2O_ (MPa)	ψ_π_ (MPa)
‘Black Beauty’	0	7.5 ± 0.6 abA	31.4 ± 0.3 bA	74.7 ± 0.3 aB	16.1 ± 0.33 aB	0.78 ± 0.0 aA	−0.5 ± 0.03 aA	−0.6 ± 0.04 aA
	40	8.5 ± 0.9 aB	37.0 ± 0.5 aA	27.9 ± 0.2 bB	7.1 ± 0.05 bB	0.74 ± 0.0 aA	−0.9 ± 0.10 abB	−1.2 ± 0.15 abB
	80	6.5 ± 0.8 bB	22.9 ± 0.6 cB	14.5 ± 0.4 cB	5.4 ± 0.12 cB	0.62 ± 0.01 bB	−1.4 ± 0.09 bB	−1.5 ± 0.04 bB
	160	5.4 ± 0.6 cB	18.4 ± 0.6 dB	9.1b ± 0.3 dB	4.4 ± 0.06 dB	0.50 ± 0.02 cB	−1.9 ± 0.19 cB	−2.5 ± 0.46 cB
‘Bonica’	0	9.9 ± 0.8 abA	34.7 ± 0.8 bA	149.8 ± 0.4 aA	25.9 ± 0.34 aA	0.82 ± 0.0 aA	−0.5 ± 0.03 aA	−0.6 ± 0.04 aA
	40	10.7 ± 0.7 aA	38.0 ± 0.9 aA	113.1 ± 0.9 bA	19.6 ± 0.24 bA	0.82 ± 0.0 aA	−0.6 ± 0.04 aA	−0.6 ± 0.08 aA
	80	9.0 ± 1.2 bA	29.5 ± 0.8 cA	103.7 ± 0.7 cA	18.0 ± 0.04 cA	0.82 ± 0.0 aA	−0.6 ± 0. 02 aA	−0.6 ± 0.04 aA
	160	8.2 ± 1.1 cA	22.7 ± 0.7 dA	94.4 ± 0.3 dA	16.6 ± 0.21 cA	0.82 ± 0.0 aA	−0.5 ± 0.02 aA	−0.7 ± 0.05 aA

Values are means ± standard errors of four replicates. Significant dissimilarities are shown by different lower-case letters between treatments and by different capital letters between varieties (*p* = 0.05) based on Tukey’s HSD test within each column and cultivar.

## Data Availability

No new data were created or analyzed in this study. Data sharing is not applicable to this article.
